# Long COVID: Clinical Framing, Biomarkers, and Therapeutic Approaches

**DOI:** 10.3390/jpm13020334

**Published:** 2023-02-15

**Authors:** Valeria Conti, Graziamaria Corbi, Francesco Sabbatino, Domenico De Pascale, Carmine Sellitto, Berenice Stefanelli, Nicola Bertini, Matteo De Simone, Luigi Liguori, Ilenia Di Paola, Maddalena De Bernardo, Angela Tesse, Nicola Rosa, Pasquale Pagliano, Amelia Filippelli

**Affiliations:** 1 Department of Medicine, Surgery and Dentistry “Scuola Medica Salernitana”, University of Salerno, Via S. Allende, 84081 Baronissi, Italy; 2 Clinical Pharmacology and Pharmacogenetics Unit, University Hospital “San Giovanni di Dio e Ruggi, D’Aragona”, 84131 Salerno, Italy; 3 Department of Translational Medical Sciences, University of Naples “Federico II”, 80131 Naples, Italy; 4 Department of Clinical Medicine and Surgery, University of Naples “Federico II”, 80131 Naples, Italy; 5 CNRS, INSERM, L’institut du Thorax, Université de Nantes, F-44000 Nantes, France

**Keywords:** COVID-19, post-COVID-19 syndrome, gender, biomarkers, phytotherapeutics

## Abstract

More than two years after the onset of the COVID-19 pandemic, healthcare providers are facing an emergency within an emergency, the so-called long COVID or post-COVID-19 syndrome (PCS). Patients diagnosed with PCS develop an extended range of persistent symptoms and/or complications from COVID-19. The risk factors and clinical manifestations are many and various. Advanced age, sex/gender, and pre-existing conditions certainly influence the pathogenesis and course of this syndrome. However, the absence of precise diagnostic and prognostic biomarkers may further complicate the clinical management of patients. This review aimed to summarize recent evidence on the factors influencing PCS, possible biomarkers, and therapeutic approaches. Older patients recovered approximately one month earlier than younger patients, with higher rates of symptoms. Fatigue during the acute phase of COVID-19 appears to be an important risk factor for symptom persistence. Female sex, older age, and active smoking are associated with a higher risk of developing PCS. The incidence of cognitive decline and the risk of death are higher in PCS patients than in controls. Complementary and alternative medicine appears to be associated with improvement in symptoms, particularly fatigue. The heterogeneous nature of post-COVID symptoms and the complexity of patients with PCS, who are often polytreated due to concomitant clinical conditions, suggest a holistic and integrated approach to provide useful guidance for the treatment and overall management of long COVID.

## 1. Introduction

In January 2020, the World Health Organization (WHO) declared a public health emergency of international significance for the 2019 coronavirus disease (COVID-19) outbreak, caused by severe acute respiratory syndrome coronavirus 2 (SARS-CoV-2) [1]. The disease reached a global pandemic status in March 2020 [2]. Since its inception, the pandemic has posed a daunting challenge, mainly because of its exponential behavior [3,4] and the absence of therapy [5]. Both repurposed and experimental drugs have been used to treat patients, often without evidence of clinical efficacy, leading to inappropriate treatment, especially in patients with comorbidities that are therefore poly-treated [5]. Luckily, several molecules have been approved and proven effective in both mild and severe/critical patients [6,7,8,9,10,11]. SARS-CoV-2 is a positive-sense single-stranded RNA virus that uses angiotensin-converting enzyme 2 (ACE2) receptors to enter the cell [12,13]. Because ACE2 is expressed in different body tissues [14], multiorgan lesions have been observed in COVID-19 [15]. Indeed, COVID-19 is a syndrome that encompasses a plethora of clinical manifestations ranging from flu-like symptoms to acute respiratory distress syndrome (ARDS) and multiple organ dysfunction syndrome (MODS) [16,17]. 

More than two years after the start of the pandemic, healthcare providers are facing an emergency within an emergency, the so-called long COVID or post-COVID-19 syndrome (PCS) [18]. According to the WHO, “post-COVID-19 condition, commonly known as long COVID, can affect anyone exposed to SARS-CoV-2, regardless of age or severity of original symptoms” [19]. It is defined as the continuation or development of new symptoms 3 months after the initial SARS-CoV-2 infection, with these symptoms lasting for at least 2 months without any other explanation [19]. 

This study aimed to review the current knowledge on the risk factors and characteristics of long COVID syndrome, focusing on describing the differences and similarities in patients of different ages, sex, and comorbid conditions. An update on biomarkers useful for diagnosing and identifying long COVID and possible therapeutic approaches was also provided.

## 2. Clinical Framing of Long COVID

Given the complexity of long COVID (or PCS), attempts to delineate and classify risk factors, signs, and symptoms have been unsuccessful [20,21]. Michelen et al. systematically reviewed 39 studies revealing considerable diversity in the reported risk factors. The authors attributed this multiplicity to the inequality of the studies published to date, which differ in design, sample size of enrolled population, and follow-up, which prevents a true understanding of this syndrome [20]. In addition to the undoubted inconsistency of studies, the multiplicity and variety of sequelae related to COVID-19 make the precise framing of PCS very complicated. Indeed, the list of PCS-related symptoms affecting any body system is extensive [22,23]. Neurological symptoms, such as fatigue and headache, are the most common, but a wide range of long-term side effects have been described, highlighting the complexity of PCS [24]. Yan et al. stressed the importance of considering that several unresolved clinical manifestations involving the respiratory, cardiovascular, neurological, and other systems may occur in COVID-19 survivors [25]. One of the trickiest aspects of long COVID is that it can occur regardless of the severity of the illness previously suffered by the subject [21]. In fact, although many cases concerned hospitalized patients, the long-term effects of COVID-19 have been described in mild disease and even in asymptomatic SARS-CoV-2 infected individuals [21]. PCS assumes particularly significant dimensions when one considers that less than 1% of COVID-19 survivors achieved complete recovery at 80 days [24], and 30% of non-hospitalized patients report persistent symptoms 2 months after the onset of SARS-COV2 infection [26].

Notably, more than 70% of survivors at low mortality risk for COVID-19 may report the persistence of impairment in one or more organs 4 months after the manifestation of initial symptoms [22]. These findings underscore the enormous impact that PCS can have on health systems and the urgency of planning appropriate countermeasures.

Although the long-term effects of COVID-19 can occur regardless of age and pre-existing medical conditions, advanced age is undoubtedly one of the most important risk factors [27].

## 3. Long COVID in the Elderly

Although older adults constitute a large proportion of COVID-19 severely affected individuals, little is known about the prevalence and risk factors of PCS among this population. The diversity of SARS-CoV-2 attack sites could explain the variety of acute, subacute, and chronic symptoms. The impact of these symptoms on autonomy and participation in social life plays an important role in both young and old people. However, while in younger patients the symptoms are mostly defined, in geriatric individuals they are quite different. In fact, the concomitance of different diseases and the coexistence of geriatric syndromes, as well as the atypia of presentation, make symptoms heterogeneous and nonspecific. This may lead to a delay in the identification and appropriate treatment of COVID-related disorders. Table 1 summarizes the main findings of long COVID studies in the elderly.

In a multicenter prospective cohort study, Daitch et al. [28] compared the symptoms of long-COVID between older (age > 65 years) and younger (18–65 years) individuals. In the study, 2333 individuals were evaluated at an average of 5 months [146 days (95% CI 142–150)] from the onset of COVID-19. The mean age was 51 years, and 20.5% were >65 years. Older participants had gone to the recovery clinic about one month earlier than younger participants (*p* < 0.001) and had higher rates of symptoms (80.0% of older subjects reported any symptoms, compared with 64.2% of younger subjects, *p* < 0.001). However, they had similar rates of high symptom burden (34.1% in older subjects vs. 32.8% in younger subjects, *p* = 0.678). Fatigue and dyspnea were the most common long COVID symptoms in both age groups (fatigue: 38.7% among older individuals vs. 39.4% among younger individuals, *p* = 0.779; dyspnea: 29.9% in older individuals vs. 27.3% in younger individuals, *p* = 0.251). Headache, chest pain, palpitations, concentration impairment, and emotional distress were all more common in the younger age group, while cough and arthralgia were more common in older adults. Older participants were more likely to have abnormal chest imaging at the time of assessment (23.2% vs. 10.1%, *p* = 0.001) and to have impairments in pulmonary function tests. The authors suggested that the higher burden of persistent symptoms among the elderly likely reflects higher rates of severe COVID-19 and subsequent hospitalizations and complications. These factors are likely to contribute to the deconditioning of the elderly population [29] added to the baseline risk for long COVID among recoverees, leading to higher proportions of symptoms.

Tosato et al. [30] studied the condition of 165 subjects and suggested that in older adults, the presence of more symptoms during the acute phase of COVID-19 is associated with a higher risk of symptom persistence beyond 2 months after hospital discharge. The presence of fatigue at the time of acute COVID-19 is a major risk factor for symptom persistence. As expected, the longer the time since acute COVID-19, the greater the likelihood of recovery from all COVID-19-related symptoms.

Differences in long COVID manifestations between older and younger adults may also reflect differences in baseline conditions, such as comorbidities, which are significantly more prevalent in the elderly. Although age and comorbidities are associated with COVID-19 severity, their impact on PCS may extend beyond the acute phase. For example, the worse age-adjusted lung function found in Daitch’s study may be explained by the decrease in lung reserve with age [31], which likely prolongs health restoration after COVID-19.

Furthermore, sarcopenia has been hypothesized as one of the factors contributing to the long duration of COVID-19 [32]. The acute inflammatory response to this infection, which includes marked elevation of inflammatory markers to the level of cytokine storm, has a high potential to harm a broad spectrum of organs and systems [33]. Clinical observation of 209 patients indicates that during the acute phase of infection, which lasts about 2 weeks, the patient is likely to lose 5–10% of body weight [34]. Sarcopenia can have a major impact on patients’ in-hospital prognosis and vulnerability to functional and physical deterioration post-COVID-19 [35]. In a cross-sectional study of 41 post-COVID-19 recoveries, Paneroni et al. found that the strength of the biceps brachii and quadriceps femoris were 69 and 54% of the predicted normal value in 73 and 86% of patients, respectively. The functionality of these large muscle groups was equally impaired. The mean age of the patients was 67.1 ±11.6 years, with a higher prevalence of men (61%) [36]. Another study identified that peak femoral blood flow was drastically reduced in 22 frail elderly patients at about 100 days after hospitalization for COVID-19 pneumonia, recognizing the altered vasculature as a component of PCS [37].

In particular, disease-related immobility has an extremely negative impact on the functionality of elderly patients. Previous studies on in-hospital immobility of elderly patients have reported muscle atrophy of about 3% per day if mobility is severely reduced [38]. While young people can compensate for this reduction relatively quickly, this is not the case for the elderly. This leads to an increased risk of falls and fractures. Persistent loss of strength also promotes permanent mobility disorders, which are associated with a loss of independence and quality of life. The inflammatory cytokine storm has been proposed to underscore this process. In particular, the inflammatory reaction caused by COVID-19 has been related to metabolic stress and muscle catabolism [39,40]. However, as suggested by Wang and colleagues, the interaction between COVID-19 and sarcopenia could be bidirectional and trigger a vicious circle [41]. Indeed, the condition of sarcopenia is known to be associated with chronic inflammation, malnutrition, metabolic and endocrine dysregulation, and various systemic dysfunctions, thus representing a key risk factor in the increased vulnerability of the elderly to COVID-19 [42].

In fact, the chronic state of low-grade inflammation that accompanies aging (inflammaging) might predispose older adults to severe COVID-19, and also contribute to symptom persistence following acute COVID-19 [43]. Inflammaging plays a pivotal role in most geriatric conditions, such as sarcopenia, disability, and multimorbidity, and is a major risk factor for several age-associated chronic diseases (e.g., cardiovascular disease, metabolic syndrome, diabetes, autoimmune disorders, dementia) [44,45,46], all of which are linked to increased COVID-19 fatality [47].

Okazaki and colleagues observed that sarcopenia is a risk factor for aspiration pneumonia in the elderly population due to dysfunction of the swallowing muscles, worsening the condition of bedridden patients with SARS-CoV-2 infection [48]. Therefore, it is to be expected that sarcopenic patients have higher infection and mortality rates in association with greater disease severity.

Among the possible damage caused by the cytokine storm, elevated C-reactive protein (CRP) concentration and subsequent mitochondrial damage have also been correlated with the onset of sarcopenia and frailty in COVID-19 patients [49]. In fact, a role for systemic inflammation has also been proposed in the involvement of bone and joint tissue in infected patients, although the underlying mechanisms are still largely unknown.

Moreover, mitochondrial damage has been recognized as having a high potential of inducing sarcopenia. Ferritin, an acute phase reactant and the key player in iron homeostasis, may directly interact with the energy production of the mitochondria [50], pushing the energy production from aerobic to anaerobic modes, enhancing radical oxygen species (ROS) generation, and increasing cellular susceptibility to damage and cell death. 

In a retrospective cohort study [51] of 1,755 older adults hospitalized with COVID-19 (mean age 75.3 years) without pre-COVID dementia, 223 (12.7%) patients developed incident dementia within 1-year follow-up. Pre-COVID psychotropic medication use was associated with a higher 1-year incidence of dementia after controlling for patient demographics, characteristics, and severity of acute COVID-19 illness (OR = 3.20, 95% CI: 2.37−4.32). Regarding the mechanisms underlying the observed association between psychotropic medications and post-COVID incident dementia, based on a population of 785 patients, the authors suggested that psychotropic medications may potentiate the neurostructural changes present in subjects who have recovered from COVID-19 [52]. The sensitivity analysis in patients with documented neurological and psychiatric diagnoses supports this interpretation. As they are not mutually exclusive, COVID-19 may have accelerated the underlying brain disorders for which psychotropic medications were prescribed, leading to the greater incidence of post-COVID dementia. 

In addition to age, sex is another variable that appears to be associated with long COVID even independently of other factors [53].

**Table 1:** Studies of long COVID in the elderly.

Source	Study Type	Country	Primary Aim	Sex (% Male)	Age Range (Years)	Total Pts (n)	Results
Daitch et al. [28]	Prospective cohort	Israel, Switzerland, Spain, Italy	To estimate the prevalence of long-COVID symptoms among older adults and to explore independent risk factors for fatigue and dyspnea.	50.8	35–66	2333	Older patients had gone to the recovery clinic about one month earlier than younger participants (*p* < 0.001) and had higher rates of any symptoms (80.0% vs. 64.2%, *p* < 0.001). However, age was not an independent predictor of fatigue and dyspnea.
Tosato et al. [30]	Cross-sectional	Italy	To provide multidisciplinary and individualized follow-up for COVID-19 survivors in the elderly.	61.6	67–80	165	The presence of more symptoms during the acute phase is associated with a higher risk of symptom persistence beyond 2 months after hospital discharge. The presence of fatigue is a major risk factor for symptom persistence.
Martincheck et al. [34]	Observational retrospective	US	To evaluate the outcome of a COVID-19 outbreak on the weight of older residents in a nursing facility.	44.0	63–86	209	During the acute phase of infection, which lasts about 2 weeks, the patients are likely to lose 5–10% of body weight.
Paneroni et al. [36]	Cross-sectional	Italy	To evaluate skeletal muscle strength, exercise intolerance, and symptoms in a cohort of patients recovering from COVID-19 pneumonia without preexisting disabilities.	61.0	40–88	41	The strength of the biceps brachii and quadriceps femoris were 69 and 54% of the predicted normal value in 73 and 86% of patients, respectively. The functionality of these large muscle groups was equally impaired.
Paneroni et al. [37]	Cross-sectional comparative study	Italy	To evaluate whether frail elderly recovering from COVID-19 pneumonia have altered vascular endothelium-dependent responsiveness.	73.0	57–78	22	The peak femoral blood flow was reduced in frail elderly patients at about 100 days after hospitalization for COVID-19 pneumonia
Douaud et al. [52]	Case control	UK	To investigate the relationship between the use of psychotropic medications and post-COVID incident dementia.	42.9	51.3–81.4	785	Pre-COVID psychotropic medication use was associated with a higher 1-year incidence of dementia, after controlling for patient demographics, characteristics, and severity of acute COVID-19 illness (OR = 3.20, 95% CI: 2.37/4.32).

PCS, Post-COVID-19 syndrome; US, United States; UK, United Kingdom; OR, odds ratio; CI, confidence interval.

## 4. Sex-Related Differences in Long-COVID

Women with COVID-19 have less severe short-term complications but suffer significant long-term sequelae [54].

A possible association between sex and long COVID has been reported in several studies. Table 2 summarizes the main findings on sex-related differences found in patients with long COVID. 

In a prospective cohort study conducted at San Paolo Hospital in Milan, Italy, 377 patients hospitalized for COVID-19 were evaluated at 1–3 months after discharge. Of these, 260 patients experienced PCS, and the majority (81.7%) was women. The most frequently reported symptoms were fatigue (39.5%) and dyspnea (28.9%), followed by musculoskeletal pain and cognitive dulling (brain fog). Anxiety, followed by depressive symptoms, was also frequently reported [55].

The multicenter cohort LONG-COVID-EXP-CM study analyzed the impact of sex on symptoms related to COVID-19 and PCS. The study was conducted in five Spanish public hospitals and included 1969 participants randomly selected from a list of 7150 patients hospitalized for COVID-19 during the first wave of the pandemic. All subjects were interviewed approximately 8 months after discharge and asked to report any symptoms that arose after discharge from the hospital. The number of post-COVID manifestations was higher in women than men (*p* < 0.001), and women were more likely to have three or more symptoms (*p* < 0.001), particularly dyspnea, pain, hair loss and eye problems, and anxiety and deterioration of sleep quality [56].

Another study, carried out by the International Severe Acute Respiratory and Emerging Infections Consortium (ISARIC) [57], tried to establish the long-term effects of COVID-19 following hospitalization. Of 327 recruited subjects, 135 (41.3%) were women. Compared by gender, the majority of the population were women who did not feel fully recovered (56.3%). Females under the age of 50 years were five times less likely to report feeling recovered (adjusted OR 5.09, 95% CI 1.64 to 15.74), were more likely to have a greater disability (adjusted OR 4.22, 95% CI 1.12 to 15.94), twice as likely to report worse fatigue (adjusted OR 2.06, 95% CI 0.81 to 3.31), and seven times more likely to become more breathless (adjusted OR 7.15, 95% CI 2.24 to 22.83) than men of the same age.

According to data from 2649 patients discharged from four hospitals in Moscow, 1247 participants (47.1%) reported persistent dermatological, respiratory, and neurological symptoms as well as mood and behavioral alterations. Notably, the female sex was associated with all of these manifestations [53]. Moreover, women are not only more exposed to PCS, but the time of symptom persistence is longer compared with men. In a large cross-sectional study that enrolled 3972 participants, Robineau et al. showed that of 861 patients with persistent symptoms, 75.4% were women, and the female sex was also associated with slower resolution of anosmia, ageusia, and asthenia [58].

Several pathophysiological mechanisms have been hypothesized to explain long COVID from the perspective of sex and gender, but they have been only partially verified. There is strong evidence that, in the context of viral infections, sexual dimorphism plays a central role in the genetic and hormonal regulation of both the innate and adaptive immune system [59]. Indeed, women have stronger innate and adaptive immune system reactivity than men [60], and as a result, they are also more susceptible to immune diseases and respond differently than men to anti-inflammatory and immunosuppressive drugs [60,61].

The hypothesis that PCS may be an estrogen-associated autoimmune disease [62] fits with the fact that women have twice the risk of developing such a syndrome than men, but when considering patients older than 60 years, this risk becomes similar in both sexes [55].

In conclusion, there is consistent evidence that women are particularly susceptible to long COVID compared with men, especially between the ages of 40 and 60 years, likely due to different effects of risk factors on survival [63]. Although the available data need to be confirmed, they stimulated the study of mechanisms and therapeutic targets for women’s overall health.

Regardless of age and sex, given the social burden attributable to long COVID, it is urgent to characterize the manifestations of this illness in all affected individuals.

## 5. Long-COVID Neurological and Psychological Symptoms

A recent meta-analysis identified 55 long-term effects of COVID-19, most of which involved the central or peripheral nervous system [24]. A cross-sectional study recruited 1539 COVID-19 patients older than 60 years discharged from COVID-19 hospitals in Wuhan, China, in 2020. Increased cognitive impairment was reported in severe COVID-19 patients, where COVID-19 severity, delirium, and chronic obstructive pulmonary disease were considered risk factors [64]. Neurological symptoms, including anosmia, ageusia, fatigue, and cognitive impairment, have been frequently reported in patients manifesting COVID-19 sequelae [64]. A study, which evaluated 3233 patients aged 60 years and older discharged from 3 COVID-19-designated hospitals in Wuhan, China from February 10 to 10 April 2020 demonstrated a higher incidence of cognitive decline compared with uninfected controls [65]. Cognitive dysfunction can be observed in many brain domains, as assessed by an online survey of more than 3000 patients with suspected or confirmed COVID-19 in the United States during a 7-week period from September 2020. Eighty-five percent of patients reported that brain fog/cognitive dysfunction peaked 4 months after the onset of COVID-19 symptoms, as well as impairments in memory, attention, executive functioning, problem-solving, and decision-making [66]. Based on a large meta-analysis that included more than 25,000 patients, no difference was observed in the incidence of fatigue and cognitive impairment between those who required or did not require hospitalization. Thirty-two percent of patients, mainly adults compared with children, experienced fatigue for more than 12 weeks after the diagnosis of COVID-19. The same study found that the percentage of subjects who had cognitive impairment was 22%, with no difference in hospitalization, and a slightly nonsignificant prevalence among women [67].

In another observational study that enrolled 242 patients, Frontera et al. evaluated more than 100 patients reporting clinical manifestations more than 3 months after the diagnosis of COVID-19 and identified three main symptom groups, one with continuous symptoms, most commonly headache, one with many relevant symptoms, including high levels of anxiety and depression, and one reporting shortness of breath, headache, and cognitive symptoms [68]. In a cohort study, a higher prevalence of self-reported memory problems was found at eight months after SARS-CoV-2 positivity than in the control group represented by untested or negative test subjects [69]. In addition, among 81 patients, Rass et al. found a prevalence of memory and concentration difficulties of 23% and 18% at 3 months and 1 year after acute COVID-19, respectively [70]. These results, although not conclusive, are not to be underestimated, considering that memory loss may increase the risk of developing neurodegenerative conditions such as Alzheimer’s disease (COVID-19 as a risk factor for Alzheimer’s disease), as also suggested by the higher serum amyloid levels in survivors 3 months after COVID-19 diagnosis found in 72 patients [71]. Although neuroinvasion of SARS-CoV-2 has not been demonstrated, prolonged inflammation may impact neurogenesis, neurotransmission, and neuroinflammation. Syrian golden hamster model showed that after SARS-CoV-2 infection, viral RNA was detected only in the olfactory neuroepithelium, whereas no viral RNA was found in the hippocampus or medulla oblongata. At the same time, destruction of the blood–brain barrier was detected in the first days of infection, resulting in the activation of microglia and the production of inflammatory cytokines. This process results in a decrease in the number of cells expressing Ki67, a marker of proliferation, and DCX, a marker of neuroblasts and immature neurons, which can be observed in hippocampal areas. Based on these data, we can speculate that the persistence of these processes results in prolonged neuropsychiatric symptoms belonging to PCS [72]. 

In addition, it has been shown in 12 patients that functional connectivity is reduced in patients with COVID-induced disorder of consciousness (DoC) compared with healthy controls. Furthermore, white matter integrity was found to be reduced in subjects with COVID-DoC compared with healthy controls and was comparable between patients with COVID-DoC and those with DoC caused by severe traumatic brain injury. Since both microhemorrhages and leukoencephalopathy were observed in several patients, it is possible that these findings represent manifestations of neurological injury present in all patients with COVID-DoC [73].

Several studies have described a post-COVID-19 chronic fatigue syndrome, which is characterized by fatigue and lack of energy in patients with Parkinson’s disease (PD). In these individuals, COVID-19 may increase the risk of delirium and worsening pain, anxiety, and depression in part due to limitations in cognitive stimulus and restrictions in mobility and social interactions [74]. This is not surprising, considering that anxiety and depression, particularly in advanced stages of PD, are common consequences of prolonged shielding, staying indoors, and lack of exercise [74]. Most PD patients with COVID-19 present with musculoskeletal pain. The new onset of sleep disturbances could further contribute to the secondary worsening of motor and non-motor symptoms. Insomnia, sleep behavior disorders due to rapid eye movement (RBD), and excessive daytime sleepiness could be part of the PCS of PD patients [75]. Furthermore, ocular complications have emerged as another manifestation of PCS, possibly arising from the neurotropism of SARS-CoV-2 [76]. Indeed, it is known that viral agents can cause a direct cytopathic effect on retinal neural cells [77,78]. This finding, together with the already-demonstrated expression of the ACE2 receptor on retinal cells [79], may explain the detection of SARS-CoV-2 in retinal biopsies obtained from patients who died from COVID-19 [80]. Indeed, the incidence of ocular disease was higher after the pandemic than before, suggesting a relationship between SARS-CoV-2 infection and long-term ocular complications. Costa et al. reported higher intraocular pressure and discrete changes in the outer retina, but no signs of uveitis in COVID-19 survivors, assessed at a mean time of 82 ± 36.4 days from disease onset [81]. 

Neurotropism of SARS-CoV-2 could explain the ophthalmic and neuro-ophthalmic manifestations described during the pandemic [76,82]. For example, Ortiz-Seller et al. [76] reported a case of a COVID-19 patient diagnosed with inflammatory chorioretinopathy and Adie’s tonic pupil. The latter condition is due to damage to postganglionic nerve fibers, which innervate the pupillary sphincter and ciliary muscle, possibly attributable to SARS-CoV-2 neurotropism. Tohamy et al. [83] retrospectively assessed ocular manifestations such as retinal vascular occlusion, anterior ischemic optic neuropathy, uveitis, and central serous chorioretinopathy in 100 post-acute COVID-19 patients. The authors found that COVID-19 survivors had greater ocular morbidity than controls. In addition, because D-dimer and erythrocyte sedimentation rates (ESR) were the only laboratory parameters that were higher in these patients compared with controls, the authors concluded that ocular comorbidities could be related to systemic inflammation related to SARS-CoV-2 infection, particularly coagulation alterations. Jossy et al. [84] described three patients who developed optic neuritis while recovering from COVID-19 infection. One was positive for serum antigens. One patient was positive for serum antibodies to myelin oligodendrocyte glycoprotein (MOG), and the other two had demyelinating lesions. All patients had vision loss during the recovery period from the infection. Two of them developed ocular symptoms and signs in the first six weeks and one patient six months after recovery. Bitirgen et al. [85] analyzed 35 subjects with previous COVID-19 and 30 healthy controls participating in a cross-sectional comparative study. In the COVID-19 group, dynamic pupillometry tests revealed significant alterations in luminal pupillary contractile responses, indicative of parasympathetic dysfunction. Again, this finding could be attributed to small fiber neuropathy related to SARS-CoV-2 infection and its neurotropism. These data highlight that the eye plays a role in coronavirus transmission and that the long-term effects of COVID-19 on ocular manifestations should be carefully considered by planning detailed follow-up of COVID-19 survivors. 

Unfortunately, the long-term effects of COVID-19 may worsen pre-existing conditions, as already reported for PD patients. This particularly concerns cancer, because the chronic pro-inflammatory state that characterizes COVID-19 can both worsen the clinical condition of cancer patients and facilitate cancer susceptibility and development. 

Table 3 reports the main findings concerning neurological and psychological symptoms associated with PCS.

## 6. Long COVID and Cancer

Along with the increasing number of patients with COVID-19 sequelae, there is an urgent need to investigate the potential link between this syndrome and cancer. Here, we focused on two main aspects: (i) the characteristics and effects of long COVID-19 in cancer patients and (ii) the biological mechanisms involved in the pathogenesis of long COVID, which could potentially enhance cancer susceptibility and development. Over the past two years, a great deal of evidence has been published about the impact of acute COVID-19 on cancer patients. Several lines of evidence have shown that cancer patients are highly vulnerable to COVID-19 and its complications. These include admission to an intensive care unit (ICU), the need for mechanical ventilation (MV), and death [86,87,88,89,90]. The estimated mortality rate from COVID-19 in cancer patients reaches 25–30% [86,87,88,89,90]. However, while the short-term complications of COVID-19 seem to be well-defined, few lines of evidence underlie and define the potential impact that prolonged COVID-19 infection might exert on cancer pathogenesis and prognosis in cancer patients. Pinato et al. demonstrated that up to 15% of 1557 cancer patients have sequelae of COVID-19, impacting both survival outcomes and compliance with cancer-specific treatments [91]. In this retrospective study, patients with a history of solid or hematologic cancer (active or in remission at the time of COVID-19 diagnosis) were included in the study population. Approximately 15% of patients showed long-term effects associated with sex (men versus women, *p* = 0.041), age (elderly versus younger patients, *p* = 0.048), number of comorbidities (two or more versus one or none, *p* = 0.0006), and smoking status (current or former smokers versus nonsmokers, *p* = 0.0004). The risk of death from sequelae of COVID-19 in these patients was also increased compared with noncancer patients (HR 1.80 [95%CI 1.18–2.75]). Finally, cancer treatment was also affected by permanent discontinuation and dose/regimen adjustment of therapy reported in 15% and 38.2% of patients, respectively. Accordingly, in cancer patients with COVID-19 sequelae, permanent treatment discontinuation was associated with an increased risk of death (HR 3.53 [95%CI 1.45–8.59]) [91]. 

Dagher et al. showed that long COVID-19 can persist up to 14 months after acute disease and occurs in up to 60% of 312 cancer patients. Post-COVID-19 effects included fatigue, sleep disturbances, myalgia, gastrointestinal symptoms, dyspnea, and cough in 82%, 78%, 67%, 61%, 47%, and 46%, respectively. Of note, the persistence of these symptoms was higher in women than in men (63% vs. 37%, *p* = 0.036) [92]. No association was found between cancer types and long COVID-19 sequelae. Along the same lines, Sharafeldin et al. described the clinical characteristics of 1700 cancer patients with PCS by retrospectively analyzing a large population. The most common cancer types included were cancers of the skin (21.9%), breast (17.7%), prostate (8.3%), and lymphoma (8.0%) [93]. Characteristics of patients were a median age of 64 years (range: 54–72 years), (ii) female sex (60.4%), (iii) non-Hispanic white ethnicity (76.8%), and (iv) current or former smokers (41.1%). In addition, cancer patients with long COVID had more comorbidities (OR = 4.3, [95%CI 2.9–6.2], *p* < 0.0001), and were more likely to be hospitalized for COVID-19 (OR = 1.3, [95%CI 1.0–1.7], *p* = 0.05) compared with non-cancer subjects [92]. This evidence supports the hypothesis that cancer patients are more susceptible to both COVID-19 acute and long-term complications as compared with non-cancer patients. However, the retrospective and heterogeneous nature of the available studies underscores the need for further investigation. 

While the characterization of PCS in relation to different malignancies is important, the study of the potential relationships between the effects of COVID-19 and the pathogenesis or predisposition of cancer remains an even more clinically relevant topic. So far, no evidence supports a direct link between cancer pathogenesis and COVID-19. However, several biological mechanisms could be involved in facilitating cancer development. These include (i) cytokine release, (ii) dysregulation of the T-cell immune response, (iii) dysregulation of cell signaling pathways, and (iv) alteration of the composition of the gut microbiota [89,94]. Chronic pro-inflammatory status is known to facilitate the development of cancer and tumor immune escape and promote all stages of tumorigenesis [95,96,97], and cytokine release is a major player in the pathogenesis of long COVID [98,99,100,101,102]. In this regard, it has been reported that post-COVID-19 patients develop a cytokine syndrome characterized by an imbalance of pro-inflammatory and anti-inflammatory cytokines, such as IL-17 and IL-2, and IL-10 and IL-4, compared with COVID-19 patients without sequelae [99]. This specific cytokine profile is associated with increased expression of T helper 17 (Th17) cells, which in turn are stimulated by cytokine imbalance [98,99]. Along the same lines, patients with long-standing COVID-19 have been reported to have a pro-inflammatory cytokine profile characterized by higher levels of IL-1β, IL-6, IL-13, IL-17A, TNFα, IFNγ-induced protein-10 (IP-10), and G-CSF [100,101,102]. In both cases, altered cytokine levels result in a chronic pro-inflammatory state that most likely reflects the abnormal host immune response induced by persistent virus replication, the persistent release of viral antigens, and/or dysregulation of immune cell activity [98]. However, further studies are needed to better clarify the role of long COVID in the chronic inflammation-induced state. 

In addition to pro-inflammatory status, T cell immune response dysregulation in patients with PCS may also predispose them to chronic inflammation. As a result, tumor immune escape also seems to be facilitated. Dysregulation of the T cell immune response plays an important role in the pathogenesis of long COVID-19. Indeed, emerging evidence has shown that patients with long COVID-19 are characterized by higher and more persistent levels of SARS-CoV2 antigen-specific follicular CD4+ T and T helper cells than patients with COVID-19 without sequelae [98,103]. Persistence of high levels of these immune cells may lead to their exhaustion, as suggested by overexpression of the checkpoint molecule programmed cell death-1 (PD-1) and its ligand PD-L1. The latter are well known to be involved in T cell depletion markers and inhibition of host anti-tumor immune response [104,105,106], facilitating cancer immune escape. In this regard, anti-PD-1/PD-L1-based immunotherapy represents a milestone in clinical oncology for the treatment of various types of solid and hematologic cancers [107,108,109]. Loretelli et al. suggested that ex vivo blockade of PD-1 can reverse T cell depletion and restore their functions in patients with long-standing COVID-19 [104]. In addition to the involvement of CD4+ T cells and follicular helper T cells, other studies have shown that in patients with long-standing COVID-19, there is a remodeling of the CD8+ T cell profile, characterized by a reduction in CD107a+, an increase in CD57+, and a significant decrease in the naïve T cell population [110,111]. Taken together, all this evidence suggests that long COVID may promote a state of pro-inflammatory cytokines and dysregulation of the T cell immune response by polarizing toward a state of CD4+ and CD8+ T-cell depletion [110,111]. Consequently, it seems reasonable to hypothesize a potential link between long COVID and cancer development or immune escape. 

Long COVID not only has a direct effect on immune cell dysregulation but has also been linked to dysregulation of cell signaling pathways that facilitate direct proliferation and inhibition of apoptotic induction of cancer cells. Other single-stranded RNA viruses, such as hepatitis C virus (HCV) and human T-cell lymphotropic virus type 1 (HTLV-1), are known to integrate into the host genome, promoting the carcinogenesis of liver cancer and adult T-cell leukemia/lymphoma, respectively [112,113]. These viruses can exert their oncogenic activity through several mechanisms, including the modification of signaling pathways [112,113]. In fact, SARS-CoV2 is not considered to be able to integrate into the host genome like a typical oncovirus. However, recent evidence suggests that signaling pathways known to play a role in cancer development are altered in COVID-19 patients along with SARS-CoV2 infection [94,98,114]. For example, SARS-CoV2 interacts with several targets in the mTOR pathway [98,115,116], which is often dysregulated in cancer and modulates several pro-tumorigenic processes, such as inhibition of induction of apoptosis, autophagy, metabolism, proliferation, and protein synthesis [117]. Grimes et al. demonstrated that SARS-CoV2 can cause upregulation of the p38 MAPK pathway either through direct activation of a specific viral protein or through loss of ACE2 [118]. The p38 MAPK pathway modulates several cellular processes, such as DNA damage repair (DDR) and oxidative stress response, which are often upregulated in several types of cancer [119]. Finally, Singh et al. demonstrated in silico an interaction between the S2 protein subunit of SARS-CoV2 and tumor suppressor proteins such as p53 and BRCA1/2 [98,120]. The latter plays an important role in inhibiting cancer cell development. Further studies will be needed to better define this preliminary evidence on the potential impact of long COVID alterations in cancer cell signaling pathways. 

Finally, increasing evidence has shown that the composition of the gut microbiota influences cancer susceptibility and prognosis as well as response to onco-immunological treatments in cancer patients [121,122]. This is important, considering that up to 30% of patients with long COVID may present with gastrointestinal symptoms [123] and that long COVID is associated with altered gut microbiota composition [98,124,125,126]. Altering the composition of the gut microbiota can create a vicious cycle by promoting the sequelae of COVID, which, in turn, alters the gut microbiota [98,124,125,126]. Furthermore, patients with PCS are characterized by a significant decrease in the richness and diversity of gut bacteria. However, it remains to be demonstrated whether these alterations can modify cancer cell development or response to onco-immunological treatments [98,127,128,129]. 

Table 4 summarizes the main findings on PCS in cancer patients. 

## 7. Biomarkers for Long-COVID Syndrome

Although the clinical manifestations may vary widely, the inflammatory process and dysregulation of the immune system are consistent features. Circulating levels of cytokines, hematologic abnormalities such as alterations in the coagulation cascade, and molecules involved in the innate and adaptive immune response have been proposed as valuable diagnostic and prognostic biomarkers of COVID-19 [106,130]. 

Similarly, indices of immune inflammation are altered in patients presenting long-term COVID-19 sequelae and can be used as biomarkers of PCS [131]. In this field, identification of diagnostic and prognostic biomarkers could improve clinical management. However, in the absence of randomized controlled trials (RCTs), most of this information comes from retrospective or prospective observational studies that unfortunately enrolled a small number of patients. 

Colarusso et al. suggested that lung fibrosis-like changes could be predicted by higher plasma levels of IL-1 α and TGF- β and lower plasma levels of IFN- β in 52 subjects who had suffered moderate or severe COVID-19. In addition, higher circulating levels of CRP, complement complex C5b-9, and LDH were found in these patients than in healthy volunteers [132]. In a pilot study conducted in a small cohort of 30 adolescents infected during the second wave of the pandemic, Petrella et al. found that serum NGF and BDNF could be used as early biomarkers of COVID-19 morbidity, and TGF-β could be a biomarker of long COVID in young women [133]. 

A Spanish cohort study that enrolled 30 subjects with PCS and 20 control subjects who recovered completely within 4 weeks found higher levels of functional memory cells with high antiviral cytotoxic activity in the PCS group compared with controls, such as CD8+ TEMRA cells, CD8 ± TCRgd + cells, and NK cells with CD56 + CD57 + NKG2C + phenotype, supported by higher levels of CD4+ Tregs and expression of the depletion marker PD-1 on the surface of CD3+ T lymphocytes [105]. Other proinflammatory molecules found at particularly high levels in subjects who manifested post-acute sequelae of SARS-COV-2 infection were TNF-α, interferon-γ–induced protein 10, and monocyte chemoattractant protein 1 (MCP-1).

Although IL-6 has been recognized as a marker and molecular target such as to justify the use of anti-IL-6 tocilizumab in patients with severe COVID-19 [8], in post-COVID-19 there was only a trend of increasing IL6 levels during the early phase of recovery, which became more pronounced thereafter [101]. 

PCS might be associated with the development of autoantibodies. Analyzing 116 subjects, a population of COVID-19 patients convalescing after ICU admission showed a higher percentage of autoantibodies than a COVID-19, non-hospitalized cohort (75% vs. 54%). The first group showed autoantibodies against seven autoantigens with a preponderance of epidermal (41%) and skeletal (17%) antibodies, while the second group against only four autoantigens (epidermal 25%, smooth muscle 17%, ANCA 8%, and parietal gastric 4%) [134].

As in COVID-19, endothelial modifications are associated with systemic risk and coagulation disorders in patients with PCS [135]. An Irish study that enrolled 50 subjects observed that the persistence of endotheliopathy is common in convalescent COVID-19 patients, suggesting its involvement in the pathogenesis of long COVID. The study described the statistically significant increase in endogenous and peak thrombin potential and the significant increase in endothelial cell biomarkers including VWF:Ag, VWF propeptide, Factor VIII, and soluble thrombomodulin in plasma in the study population [136]. Another finding on endotheliopathy was evaluated in a study that enrolled 30 patients with PCS with persistent fatigue and exercise intolerance and 15 healthy seronegative controls. A subgroup of patients showed endothelial dysfunction that was identified by a decrease in the reactive hyperemia index; the concentration levels of endothelin-1 were significantly elevated, while those of angiopoietin-2 were lower compared with controls [137]. 

In a multicenter study, Liu et al. performed a shotgun metagenomic sequencing to evaluate whether the fecal microbiome was correlated with persistent symptoms 6 months after recovery from COVID-19. The authors found that respiratory and neurologic symptoms were positively correlated with the presence of opportunistic and nosocomial pathogens, respectively. On the contrary, an inverse correlation between butyrate-producing bacteria and PCS was found. As previously reported in the section on long COVID in cancer patients, these findings suggest that alteration of the gut microbiota could be useful as a biomarker of PCS [138,139]. 

Given the important differences between the sexes in both COVID-19 and long COVID syndrome, one would expect to find useful biomarkers to stratify the risk of long COVID between the two sexes. However, data are currently very scarce.

In a cross-sectional study including 121 patients, Maamar et al. found that a higher neutrophil count correlated with post-COVID fatigue in men and post-COVID anosmia in young women. In addition, CRP serum level was associated with PCS in men but not in women, probably because of a higher biological response to pro-inflammatory cytokines in men than in women during COVID-19 [140].

A case-control study analyzed endothelium-dependent flow-mediated dilation (FMD) included in 133 convalescent COVID-19 patients and 133 age-matched controls. FMD was directly correlated with arterial oxygen tension, forced expiratory volume in 1 s, forced vital capacity, and carbon monoxide diffusing capacity (rho = 0.280, *p* = 0.008). COVID-19 survivors showed significantly lower FMD than controls (*p* < 0.001). Women showed significantly higher values than convalescent men (*p* < 0.001) [141].

Another case-control study on 92 subjects highlighted elevated levels of 14 blood biomarkers of vascular transformation in the group of patients with long COVID. In particular, ANG-1 and *p*-SEL, angiogenic biomarkers, showed excellent sensitivity and specificity for patients with Long-COVID (*p* < 0.0001). Moreover, ANG-1 levels were found to be associated with the female sex (*p* < 0.05) [142].

Further studies are warranted to identify biomarkers in the general population and from a sex/gender perspective to address specific and early therapeutic approaches to contrast long COVID.

## 8. Therapeutic Approaches

Given the importance of long COVID from the social point of view and its economic impact on healthcare systems, efforts are being made to draft guidelines for the management of this syndrome [143,144].

Several therapeutic approaches, both pharmacological and non-pharmacological, have been proposed. However, drug therapy is symptomatic and currently, there are no medications to treat the condition itself [143,144]. Ongoing studies of recently approved drugs for COVID-19 (e.g., paxlovid) and repurposed drugs will hopefully provide effective solutions [145]. 

A growing trend in the therapeutic management of PCS involves the use of complementary and alternative medicine (CAM). CAM has greatly increased during the last two pandemic years since vitamins, herbal medicines, and other supplements could be a cost-effective and safe option for the maintenance of health status [146] and in particular for optimal immune defense [147]. 

Indeed, recently it has been suggested that the systemic immune response reflected by antibodies to SARS-CoV-2 is strongly correlated with the severity of post-COVID fatigue [148] and that the response against the SARS-CoV-2 nucleocapsid may play a more important role than the spike in the course of long-term COVID syndrome [149], supporting the evidence that COVID-19 vaccination lowers the severity and life impact of long COVID among patients with persistent symptoms [150]. 

Several natural extracts containing bioactive compounds such as quercetin and anthocyanins have been proposed by molecular docking studies as effective anti-SARS-CoV-2 agents [151]. In this field, an in vitro study has shown that the anthocyanin-rich fraction of black rice germ and bran is able to attenuate inflammatory responses induced by the S1 subunit of the spike glycoprotein of SARS-CoV-2 through activation of NF-kB and down-regulation of inflammasome-dependent proteins (NLRP3, ASC, and caspase-1) at both gene and protein levels (*p* < 0.05) [152].

In an RCT that enrolled 188 patients and 25 controls, Kharaeva et al. showed that the use of *Carica papaya* and *Morinda citrifolia* supplements reduced post-COVID symptoms, with increased leukocyte phagocytic capacity, antioxidant activity, and ATP content [153].

Various supplement formulations have been tested in different studies, mainly to reduce neurological symptoms (e.g., fatigue, memory loss), which, as mentioned above, are the most frequently reported in PCS.

An observational study enrolling 201 subjects who took a multivitamin and mineral supplement for 28 days showed a significant reduction (*p* < 0.0001) in general fatigue, mental fatigue, and quality of life, as assessed by specific questionnaires [154].

Similarly, Naureen et al. showed that a dietary intake of a supplement containing hydroxytyrosol, acetyl L-carnitine, and B, C, and D vitamins for 15 days was effective in reducing chronic fatigue in 20 PCS patients compared with 20 controls [155]. Another RCT, enrolling 46 post-COVID patients, found that a combined L-arginine and vitamin C supplementation for 28 days could improve fatigue and endothelial dysfunction, which is commonly associated with PCS. In fact, there was an increase of 6 min walk distance (*p* = 0.001), handgrip strength (*p* = 0.03), the FMD (*p* = 0.03), and fatigue (*p* < 0.0001) in the active group compared with placebo [156]. In addition, Pycnogenol, a tannin-rich supplement, was shown to improve both endothelial function and fatigue after 3 months in 60 PCS patients [157]. 

Recently, the use of CAM has been increasing in neurology [158,159]. Indeed, supplements could delay the worsening of cognitive deterioration often reported by survivors of COVID-19. Bove et al. tested the effect of a nutraceutical supplement combined with nootropic effects on 40 elderly patients with PCS who had cognitive impairment. After 3 months, this agent significantly improved (*p* < 0.05) functional status, as assessed by the Mini-Mental State Examination (MMSE), and major psychological complaints by PSQ (Perceived Stress Questionnaire), SRDS (Self-Rating Depression Scale) [160]. So far, only the potential benefits of these supplements in COVID-19 in both acute and long-term phases have been reported in the literature. However, healthcare providers and patients must be careful when taking these supplements, as they underestimate their potential toxic effects [5]. In addition to drug–drug interactions [5], drug–supplement interactions should also be considered. Various adverse reactions, including life-threatening toxicities [161,162] up to cases of therapeutic failure [163,164] can occur in patients taking concomitantly supplements and drugs. Table 5 summarizes the main findings of the studies reporting the use of CAM in PCS.

Non-pharmacological options include physical rehabilitation programs, mental health support, and social assistance [165].

Pulmonary rehabilitation programs can improve lung functional impairment and/or persistent dyspnea and, consequently, quality of life. A longitudinal one-year follow-up study highlighted that, after a rehabilitation program, exercise dyspnea, functional assessments (e.g., 6-min walking distance), hyperventilation syndrome, and quality of life improved significantly [166]. 

Today, following these physical rehabilitation programs has certainly become easier and more convenient due to the development of telemedicine. Nevertheless, in several countries (e.g., the United Kingdom), for example, only 0.4% of COVID-19 patients joined a rehabilitation program. The scarce use of rehabilitation services could be due to the absence of diagnostic care pathways, as well as to the disallowance and intricate characterization of PCS [167]. 

## 9. Conclusions

The clinical picture of PCS is extremely complex. A variety of factors can determine or contribute to the onset of this syndrome. A substantial proportion of COVID-19 survivors with long-term effects have been found in the elderly and immunocompromised patients (e.g., cancer patients). However, all individuals may present sequelae of COVID-19, including physical and psychological symptoms that healthcare providers should learn to recognize.

Neurological (e.g., fatigue, anosmia, cognitive impairment, ocular complications) and respiratory (e.g., dyspnea) symptoms were the most frequently described manifestations, affecting patients’ quality of life and ability to be self-sufficient. Women are more prone to developing PCS and have more persistent COVID-19 symptoms than men. 

Treatment of post-COVID-19 effects could hopefully be individualized and optimized using effective biomarkers. Some molecules have been proposed for the early recognition of long COVID. However, large clinical trials are urgently needed to identify new biomarkers or test those already proposed in the general population and in specific clinical settings.

The heterogeneous nature of post-COVID symptoms, the large number of patients involved, and the need for additional monitoring in paucisymptomatic and asymptomatic patients suggest a holistic and integrated approach to provide useful guidance for the treatment and overall management of long COVID.

## Figures and Tables

**Table 2:** Studies on sex-related differences in long COVID.

Source	Study Type	Country	Primary Aim	Sex (% Male)	Age Range (Year)	Total Pts (n)	Results
Munblit et al. [53]	Longitudinal cohort	Russia	To investigate the incidence of long-term consequences in adults previously hospitalized for COVID-19 and to assess risk factors for long COVID.	48.8	46–66	2649	47.1% reported persistent dermatological, respiratory, and neurological symptoms. The female sex was associated with all of these manifestations.
Bai et al. [55]	Prospective cohort	Italy	To investigate the incidence of physical and/or psychological symptoms characterizing the long COVID in women and to determine possible predictors of long COVID.	63.7	49–68	377	206 out of 377 patients experienced PCS and 81.7% were women. The most reported symptoms were fatigue (39.5%) and dyspnea (28.9%), followed by musculoskeletal pain and cognitive dulling. Female gender, older age, and active smoking were found to be associated with a higher risk of developing long COVID.
Fernández-de-las-Peñas et al. [56]	Cohort	Spain	To investigate sex differences in post-COVID symptoms.	53.6	45–77	1969	The number of post-COVID manifestations was higher in women than men (*p* < 0.001), and women were more likely to have 3 or more symptoms (*p* < 0.001).
Sigfrid et al. [57]	Prospective cohort	UK	To establish the long-term effects of COVID-19 after hospitalization.	45.3	53.2–69.8	327	Women <50 years old were 5 times less likely to report feeling recovered (OR 5.09, 95% CI 1.64 to 15.74), were more likely to have a greater disability (OR 4.22, 95% CI 1.12 to 15.94), to report worse fatigue (adjusted OR 2.06, 95% CI 0.81 to 3.31), and to become more breathless (OR 7.15, 95% CI 2.24 to 22.83) than men of the same age.
Robineau et al. [58]	Cross-sectional	France	To describe the temporal dynamics of COVID-19 long-term effects and the factors useful to predict their resolution.	73.0	38–63	3972	Of 861 patients with persistent symptoms, 75.4% were women, and the female sex was also associated with slower resolution of anosmia, ageusia, and asthenia.

PCS, post-COVID-19 syndrome; UK, United Kingdom; OR, odds ratio; CI, confidence interval.

**Table 3:** Studies on long-COVID neurological and psychological symptoms.

Source	Study Type	Country	Primary Aim	Sex (% Male)	Age Range (Years)	Total Pts (n)	Results
Liu et al. [64]	RCT	China	To investigate the long-term effects on cognitive function and to determine related risk factors in patients who recovered from COVID-19	47.95	66–75	1539	Patients with severe COVID-19 have greater cognitive decline than patients with non-severe COVID-19. [dementia: 25 vs. 9, *p* < 0.001; MCI: 60 vs. 63, *p* < 0.001] and controls [dementia: 25 vs. 0, *p* < 0.001; MCI: 60 vs. 20, *p* < 0.001)].
Liu et al. [65]	RCT	China	To assess cognitive changes in elderly PCS patients over 1 year.	48.05	66–74	3233	Higher incidence of cognitive decline in PCS patients compared with uninfected controls. Patients with severe COVID-19 have a higher risk of early- (OR: 4.87; 95% CI, 3.30–7.20), late- (OR: 7.58; 95% CI, 3.58–16.03), and progressive-onset cognitive decline (OR: 19.00; 95% CI, 9.14–39.51) than controls.
Davis et al. [66]	Multicenter observational	56 countries	To analyze the neurological and neuropsychiatric symptoms course and severity over time in COVID-19 survivors.	19.1	>18	3762	Of 3762 patients, 85% had cognitive dysfunction that peaked 4 months after the onset of COVID-19 symptoms and memory impairment, altered attention, executive functioning, problem-solving, and decision-making.
Frontera et al. [68]	Observational	US	To identify clinically important phenotypes of patients with PCS and to assess associations of these phenotypes with their functional status and quality of life.	64	53–73	242	More than 100 patients were divided into three main symptom groups: with continuous symptoms (most commonly headache), with many relevant symptoms (including high levels of anxiety and depression); reporting shortness of breath, headache, and cognitive symptoms.
Rass et al. [70]	Observational	Austria	To describe the natural course of neurological manifestations over 1 year in PCS patients.	59	47–64	81	The prevalence of memory and concentration difficulties of 23% and 18% was found at 3 months and 1 year after acute COVID-19, respectively. The most frequent symptoms at the 1-year follow-up were fatigue, difficulty in concentration, sleep disturbance, myalgia, limb weakness, headache, altered sensitivity, and hyposmia.
Zhou et al. [71]	Observational	China	To study the clinical characteristics of patients recovered from COVID-19 at 3 months post-discharge.	19.5	51.50–64	72	Significant serum amyloid levels were found in COVID-19 survivors at 3 months.
Fischer et al. [73]	Observational	US	To describe the clinical characteristics and functional status of COVID-DoC patients at 3 and 6 months after discharge.	41.7	55–76.3	12	At 3 months, the median scores of GOSE and DRS were 3 and 7, respectively; at 6 months, the median scores of GOSE and DRS were 4 and 3. Functional and structural brain connectivity of COVID-DoC patients, undergoing MRI, were decreased compared with healthy controls, and structural connectivity was comparable to that in patients with severe TBI.

MCI, mild cognitive impairment; PCS, post-COVID syndrome; OR, odds ratio; CI, confidence interval; US, United States; DoC, disorders of consciousness; GOSE, Glasgow Outcome Scale Extended; DRS, Disability Rating Scale; MRI, magnetic resonance imaging; TBI, traumatic brain injury.

**Table 4:** Studies on Long COVID and cancer.

Source	Study Type	Country	Primary Aim	Sex (% Male)	Age (Years)	Total Pts (n)	Type of Cancer (%)	Results
Pinato et al. [91]	Retrospective registry study	Belgium, France, Germany, Italy, Spain UK	To evaluate the prevalence and impact of COVID-19 sequelae in cancer patients, following recovery from SARS-CoV-2 infection.	48.2	≥18	1557	Breast (43.7) GI (34.6) GU (40.6) Hematological (27.5) Thoracic (28.1) Others (25.5) Missing (0)	234 (15%) patients reported COVID-19 sequelae. Sequelae were associated with sex, age, number of comorbidities, and smoking status. The risk of death from sequelae in cancer patients increased compared to noncancer patients (HR 1.80 [95%CI 1.18–2.75]).
Dagher et al. [92]	Observational retrospective	US	To describe long-term effects from COVID-19 recovery in cancer patients.	NA	18–86	312	NA	Long COVID-19 can persist up to 14 months after acute disease and occurs in up to 60% of cancer patients. Long COVID-19 effects included fatigue, sleep disturbances, myalgia, gastrointestinal symptoms, dyspnea, and cough (82%, 78%, 67%, 61%, 47%, and 46%, respectively). The persistence of symptoms was higher in women than in men (63% vs. 37%, *p* = 0.036).
Sharafeldin et al. [93]	Retrospective cohort study	US	To describe characteristics of long COVID in cancer patients.	39.6	54–72	1700 (1066 controls)	Skin (21.9) Breast (17.7) Prostate (8.3) Lymphoma (8.0) Leukemia (5.7)	Characteristics were (i) median age of 64 years (range: 54–72 years), (ii) female sex (60.4%), (iii) non-Hispanic white ethnicity (76.8%), (iv) current or former smokers (41.1%), (v) more comorbidities (OR = 4.3, [95%CI 2.9–6.2], *p* < 0.0001), and (vi) more likely to be hospitalized for COVID-19 (OR = 1.3, [95%CI 1.0–1.7], *p* = 0.05) compared with non-cancer subjects.

HR, hazard ratio; CI, confidence interval; CT, chemotherapy; ET, endocrine therapy; GI, gastrointestinal; GU, genito-urinary; IT, immunotherapy; TT, targeted therapy; NA, not available; OR, odds ratio; UK, United Kingdom; US, United States.

**Table 5:** Studies on complementary and alternative medicine in long COVID.

Source	Study Type	Country	Primary Aim	Sex (% Male)	Age Range (Years)	Total Pts (n)	Tested Dietary Supplement(s)	Results
Kharaeva et al. [153]	RCT	Russia	To alleviate PCS symptoms	49.7	35–69	213 (25 controls)	Fermented *Carica papaya* 14 mg per os and fermented *Morinda citrifolia* 14 mg per os Duration: 20 days	At 20 days, self-reported clinical symptoms as well as IL-6, IL-8, and nitric oxide metabolites diminished in PCS patients receiving supplementation compared to placebo. The PMNs capacity to phagocyte, AOA, and ATP content remarkably increased in the supplemented group compared to placebo.
Rossato et al. [154]	Open-label observational trial	Italy	To improve the general health status especially the PCS-related chronic fatigue	39.3	35–62	201	Vitamins D, H, B1, B3, B7, C, E 87 mg, minerals (e.g., iron, magnesium, zinc, selenium) 204 mg, amino acids (e.g., arginine, carnitine) 1.5 g, and *Panax ginseng* and *Eleutherococcus senticosus* extracts 150 mg, once daily per os Duration: 28 days	The scores on quality of life, health status, FACIT-Fatigue and mental fatigue significantly improved after the first 14 days and at 28 days.
Naureen et al. [155]	Pilot observational study	Italy	To diminish perceived PCS-related fatigue	47.5	28–76	40 (20 controls)	Vitamin C 160 mg; acetyl-L-carnitine 150 mg; hydroxytyrosol/olive polyphenols, 100 mg; thiamine 12.5 mg; vitamin B6, 5 mg; folic acid 0.2 mg; vitamin D3 0.025 mg; and vitamin B12. 0.005 mg, once daily per os Duration: 15 days	At 15 days, self-perceived energy doubled (+123%) and fatigue and tension levels halved (−51% and −48%, respectively) in the supplemented compared with the unsupplemented group.
Tosato et al. [156]	RCT	Italy	To increase physical performance, endothelial function and decrease persistent PCS-related fatigue	34.8	50.5 (median)	46	L-arginine, 1.66 g + liposomal vitamin C, 500 mg, BID, per os Duration: 28 days	At 28 days, 6 min walk distance, handgrip strength, and FMD increased significantly while self-reported fatigue was significantly lower in PCS patients belonging to the supplemented group compared with those receiving the placebo.
Belcaro et al. [157]	Pilot observational study	Italy	To ameliorate endothelial function, microcirculation inflammatory markers, and oxidative stress in PCS patients	NA	35–70	60	*Pinus pinaster* extract, 50 mg, 3 times a day (total 150 mg), per os Duration: 90 days	FMD, reactive finger hyperemia, ankle swelling rate, and renal cortical flow velocity improved significantly in the supplemented group after 1 month and after 3 months compared with controls. High-sensitivity CRP (hs-CRP), plasma Il-6 levels and oxidative stress markers decreased significantly over 3 months in the supplemented group. Questionnaire scores on quality of life, mood and fatigue, and Karnofsky scale performance index improved significantly in the supplemented group compared with controls after 1 and 3 months.
Bove et al. [158]	Pilot observational study	Italy	To improve cognitive function and psychosocial parameters in PCS patients	NA	65–68	40	*Bacopa monnieri* extract, 320 mg; L-theanine, 100 mg; *Crocus sativus L.* extract Saffron, 30 mg; vitamin B6, 9.5 mg; vitamin D, 25 µg; copper, 2 mg; biotin, 450 µg; folic acid, 400 µg; and vitamin B12, 33 µg, 1 capsule/day per os Duration: 90 days	After 90 days, functional status, assessed by the MMSE and PCFS scale, and major psychological disorders improved significantly.

PCS, post-COVID-19 syndrome; IL-6, interleukin-6; IL-8, interleukin-8; PMNs, polymorphonuclear leukocytes; AOA, antioxidant activity; FACIT, Fatigue: Functional Assessment of Chronic Illness Therapy—Fatigue Scale; FMD, flow-mediated dilation; NA, not available; CRP, c reactive protein; MMSE, Mini-Mental State Examination; PCFS, Post-COVID-19 Functional Status; PSQ, Perceived Stress Questionnaire; SRDS, Self-Rating Depression Scale.

## Data Availability

Not applicable.
